# Piezoelectric Energy Harvester Based on LiNbO_3_ Thin Films

**DOI:** 10.3390/ma13183984

**Published:** 2020-09-09

**Authors:** Zakhar Vakulov, Andrey Geldash, Daniil Khakhulin, Marina V. Il’ina, Oleg I. Il’in, Viktor S. Klimin, Vladimir N. Dzhuplin, Boris G. Konoplev, Zhubing He, Oleg A. Ageev

**Affiliations:** 1 Federal Research Centre ‘The Southern Scientific Centre of the Russian Academy of Sciences’ (SSC RAS), 344006 Rostov-on-Don, Russia; 2 Research and Education Centre ‘Nanotechnologies’, Southern Federal University, 347922 Taganrog, Russia; ageldash@sfedu.ru (A.G.); dzhuplin@sfedu.ru (V.N.D.); ageev@sfedu.ru (O.A.A.); 3 Laboratory of Functional Nanomaterials Technology, Southern Federal University, 347922 Taganrog, Russia; dhahulin@sfedu.ru (D.K.); oiilin@sfedu.ru (O.I.I.); 4 Institute of Nanotechnologies, Electronics, and Equipment Engineering, Southern Federal University, 347922 Taganrog, Russia; mailina@sfedu.ru (M.V.I.); kliminvs@sfedu.ru (V.S.K.); kbg@sfedu.ru (B.G.K.); 5 Department of Materials Science and Engineering, Shenzhen Key Laboratory of Full Spectral Solar Electricity Generation (FSSEG), Southern University of Science and Technology, Shenzhen 518055, China; hezb@sustc.edu.cn

**Keywords:** nanoelectronics, laser ablation, lead-free ferroelectrics, carbon nanotubes, thin films, energy conversion devices

## Abstract

This paper reports the results of the influence of the energy of laser pulses during laser ablation on the morphology and electro-physical properties of LiNbO_3_ nanocrystalline films. It is found that increasing laser pulse energy from 180 to 220 mJ results in the concentration of charge carriers in LiNbO_3_ films decreasing from 8.6 × 10^15^ to 1.0 × 10^13^ cm^−3^, with the mobility of charge carriers increasing from 0.43 to 17.4 cm^2^/(V·s). In addition, experimental studies of sublayer material effects on the geometric parameters of carbon nanotubes (CNTs) are performed. It is found that the material of the lower electrode has a significant effect on the formation of CNTs. CNTs obtained at the same growth time on a sample with a Cr sublayer have a smaller diameter and a longer length compared to samples with a V sublayer. Based on the obtained results, the architecture of the energy nanogenerator is proposed. The current generated by the nanogenerator is 18 nA under mechanical stress of 600 nN. The obtained piezoelectric nanogenerator parameters are used to estimate the parameters of the hybrid-carbon-nanostructures-based piezoelectric energy converter. Obtained results are promising for the development of efficient energy converters for alternative energy devices based on lead-free ferroelectric films.

## 1. Introduction

Despite a significant improvement in the performance of computing system components, essential progress in the fabrication of high-efficiency batteries has not yet been achieved [1]. The increase in performance has led to an increase in power consumption, which leads to a reduction in battery life, significantly limits the functionality of modern electronic devices, and increases the weight and size parameters of the power supply elements [2]. In addition, battery life is limited compared to the duty cycle of the device. Replacing or recharging batteries is often ineffective and sometimes impossible. Therefore, the collection and conversion of environmental energy into electric energy is considered today as an alternative to electrochemical batteries and can be used as an autonomous energy source for portable devices and wireless sensors [3,4,5,6]. Advances in wireless technology and MEMS have made it possible to use power converters as an alternative to conventional electrochemical batteries, which are already used as power sources for portable electronics [7].

Today, there are several possible ways to obtain electrical energy from environmental energy: the use of solar energy [8], electromagnetic induction [9], electrostatic generation [10], dielectric elastomers [11], and piezoelectric materials [12]. Each of the methods can power devices with a small amount of energy. However, the complex systems based on piezoelectric materials have become attractive due to their ability to convert mechanical energy (deformation energy) into useful electrical power [13,14,15].

Energy conversion in a piezoelectric material is possible due to the existence of a local charge separation called an electric dipole [16]. When mechanical stress is applied to a piezoelectric material, a dipole deforms to generate an electric charge that can be used to supply various portable electronic devices [17,18]. This effect is used in sensing elements based on piezoelectric materials in accelerometers, microphones, strain gauges, etc. The same approach is used to generate electricity, but instead of dissipating energy, it is used to power a device.

The process of collecting energy from the environment and converting it into electrical power is called energy harvesting [19,20]. The conversion efficiency depends not only on the piezoelectric coefficients but also on the applied mechanical stress (deformation). The magnitude of the generated electric power also depends on the elastic and fracture properties of the piezoelectric material, which determine the ability to withstand deformations. The efficiency of piezoelectric energy converters can be increased by modifying the properties of the active layer, the topology of the contact system, direction of polarization, geometric parameters of the active layer, and creating additional mechanical stresses to improve adhesion and increase deformation [17]. In addition, extra attention is paid to the multi-component ferroelectric oxides and carbon nanostructures., To increase the area of energy harvesting, experimental studies were carried out to create a “skeleton” based on carbon nanotubes (CNTs), which was covered with LiNbO_3_. The use of vertically oriented CNTs makes it possible to increase the effective coverage area of LiNbO_3_ due to the greater development of the CNT surface as compared to the formation of a piezoelectric layer on a flat substrate. The parameters and geometries of CNTs can be varied by the tuning of CNTs growth regimes and by using different contact materials. The use of hybrid structures leads to withstanding significant deformation gradients, thereby increasing the conversion efficiency. Moreover, the area of the possible application of energy harvesters can be significantly expanded by using lead-free piezoelectric materials [21]. Lithium niobate (LiNbO_3_) is one of the prospective materials for piezoelectric energy harvesters because it has a high Curie temperature and does not contain lead [22]. One of the key tasks in the formation of thin multicomponent oxide films is the control of the processes that affect the preservation of the stoichiometric composition, as it determines the structural and electro-physical properties of the formed films [23].

Currently, LiNbO_3_ thin films can be obtained by following technological methods compatible with modern integrated technologies of micro- and nanoelectronics: epitaxy [24], chemical vapor deposition [25], Radio Frequencysputtering [26], pulsed laser deposition [27,28,29,30], and the sol–gel process [31].

Pulsed laser deposition (PLD) is mostly used for the growth of complex oxides [32,33,34,35] and when a particular threshold value of the laser power density is exceeded, stoichiometric growth of films is possible [36]. However, the physical mechanisms underlying this phenomenon have not yet been clarified [37]. Thus, the purpose of this work was to experimentally study the influence of laser pulse energy under the deposition of LiNbO_3_ films by PLD on their morphology and electro-physical parameters, and to evaluate the possibility of using the obtained films in piezoelectric converters based on hybrid nanostructures.

## 2. Materials and Methods

**Figure 1:**
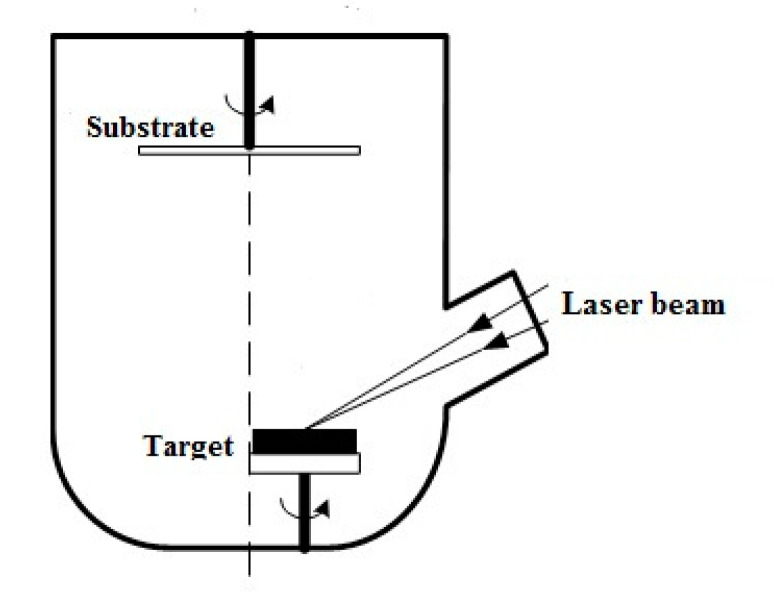
Schematic representation of the pulsed laser deposition process.

For LiNbO_3_ film formation by PLD (Figure 1), the nanotechnological cluster complex NANOFAB NTK-9 (NT-MDT, Zelenograd, Russia), with the Pioneer 180 PLD module (Neocera Co., Beltsville, MD, USA) was used. The LiNbO_3_ congruent target (Kurt J. Lasker, 99.9% purity) was ablated by excimer KrF laser (λ = 248 nm) (Coherent Inc., Santa Clara, CA, USA) with a pulse length of 20 ns. The laser pulse energy was varied in the range from 180 to 220 mJ in this case, while the target–substrate distance (80 mm), the number of pulses (50,000), the repetition rate (10 Hz), and the background oxygen pressure in the growth chamber (1 × 10^−2^ Torr) remained unchanged. LiNbO_3_ films with a thickness of 132.3 ± 8.8 nm–153.9 ± 10.5 nm were obtained at a temperature of 600 °C on single-crystal silicon substrates.

The formation of catalytic centers and the growth of carbon nanotubes (CNT) were carried out in the plasma-enhanced chemical vapor deposition (PECVD) module (NT-MDT, Zelenograd, Russia), designed for the catalytic plasma-chemical deposition of arrays of carbon nanotubes on flat plates. The deposition of CNTs took place in a plasma obtained at low pressure by means of a direct current electric discharge only on previously prepared catalytic structures with a structure suitable for CNT synthesis. Previously [38,39], we carried out the necessary studies of the growth modes of CNTs by PECVD, which made it possible to determine the optimal technological parameters of their growth. CNTs were grown on Si (100) samples coated with contact V or Cr (50 nm) and catalytic Ni layers (10 nm). The deposition was carried out in a flow of ammonia (210 sccm) and acetylene (70 sccm) at a pressure of 4.5 Torr. During the growth process, a plasma with a power of 2 W was initiated employing an electric discharge with a direct current of 5 mA at a voltage of 420 V. The gap between the electrodes was 10 mm. The range of growth times was from 1 to 10 min.

The morphology of the obtained films was studied by scanning electron microscopy (SEM) and atomic force microscopy (AFM) in semi-contact mode using a Nova Nanolab 600 scanning electron microscope (FEI. Co., Eindhoven, The Netherlands) and the Ntegra probe nanolaboratory (NT-MDT, Zelenograd, Russia), respectively [23]. The crystal structure of the obtained LiNbO_3_ films was studied by the X-ray diffraction (XRD) method using a Rigaku MiniFlex 600 (Rigaku Co., Tokyo, Japan). The charge carrier concentration and mobility were measured using the Hall effect technique by an Ecopia HMS-3000 measurement system (Ecopia Co., Anyang, Korea) [28].

## 3. Results

### 3.1. LiNbO_3_ Thin Film Growth

**Figure 2:**
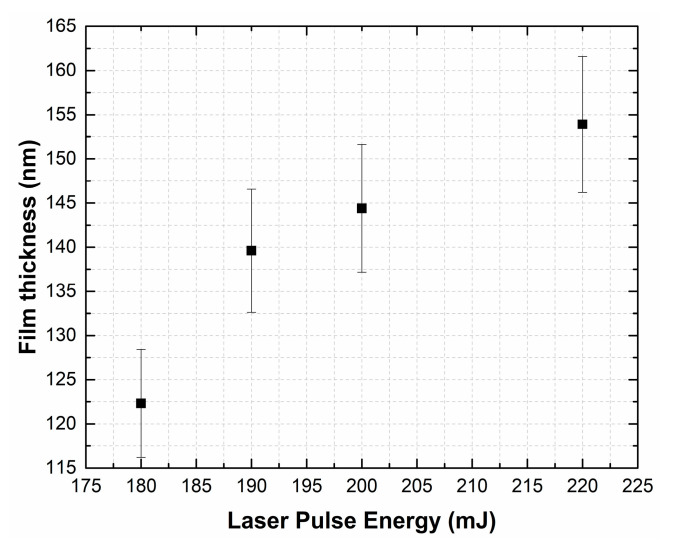
Dependence of the thickness of the obtained LiNbO_3_ films on the laser pulse energy.

Figure 2 shows the dependence of film thickness of the obtained LiNbO_3_ on laser pulse energy.

Our results indicate that with an increase in the laser pulse energy from 180 to 220 mJ, the thickness of the LiNbO_3_ films increased from 132.3 ± 8.8 nm (the deposition rate of the film was 1.47 nm/min) to 153.9 ± 10.5 nm (film deposition rate 1.71 nm/min).

**Figure 3:**
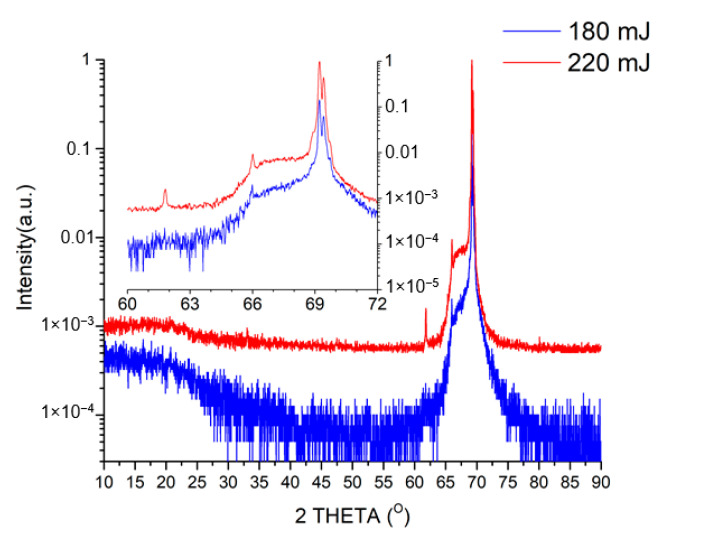
X-ray spectra of LiNbO_3_ films obtained at laser pulse energies of 180 and 220 mJ.

Figure 3 shows the X-ray diffraction patterns of films obtained at laser pulse energies of 180 and 220 mJ.

**Figure 4:**
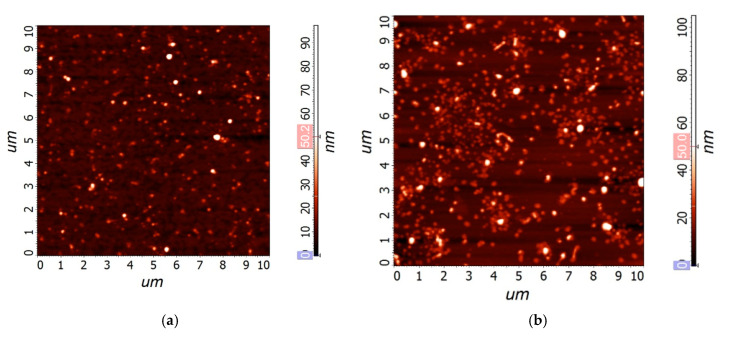
Atomic force microscopy (AFM) images of LiNbO_3_ films fabricated by pulsed laser deposition (PLD) at different laser pulse energies: 180 mJ (**a**), 200 mJ (**b**).

We can conclude that the obtained films predominantly consist of LiNbO_3_—the double peak at about 69° refers to phases (018) and (214), and the higher peak intensity in the 220 mJ sample indicates a greater film thickness obtained at a laser pulse energy of 220 mJ. The 220 mJ X-ray diffraction pattern also shows the presence of a peak corresponding to phase (211). Thus, the additional energy of the laser beam changes the mobility of ablated particles, intensifies the growth process, and leads to the formation of a thicker film with higher crystallinity. In addition, an increased number of collisions at higher energy also stimulates variation in the growth direction [37] as it is indicated by the appearance of the (211) peak. The spectra of the films are shifted to the right by ~5° relative to the spectra presented in [40,41,42]. Such a shift can be explained by stress applied to LiNbO_3_ films by Si substrates. Figure 4 shows AFM images of LiNbO_3_ films obtained at different laser pulse energies.

**Figure 5:**
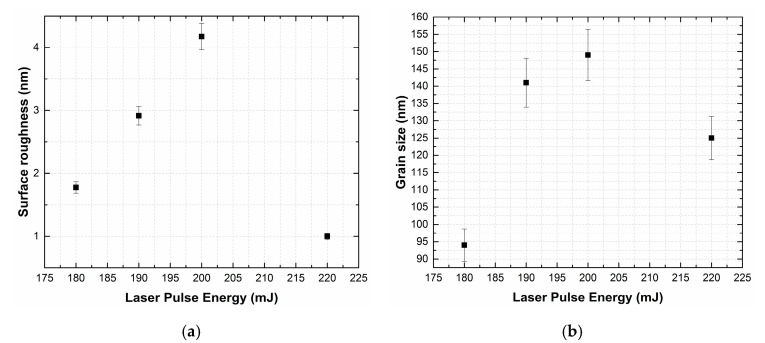
The dependencies of average surface roughness (**a**) and grain size (**b**) on laser pulse energy.

**Figure 6:**
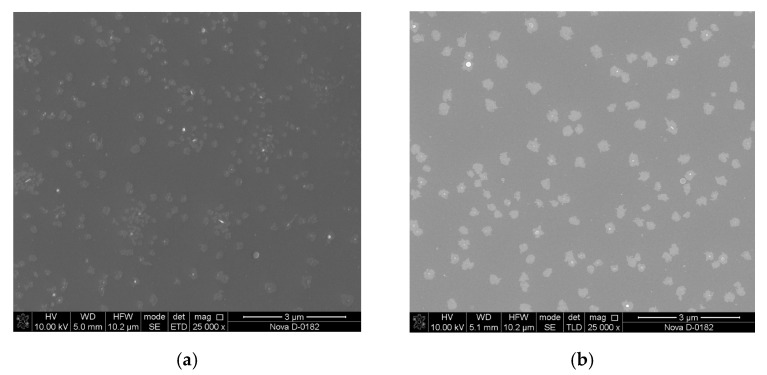
SEM images of LiNbO_3_ films fabricated with different laser pulse energies: 180 mJ (**a**) and 220 mJ (**b**).

An increase in the grain size can be related to an increase in the kinetic energy of the ablated particles and the intensification of coalescence processes (Figure 5) [37]. A further increase in the energy of laser pulses above 200 mJ leads to the formation of smaller ablated particles, which results in the formation of a fine-grained film (Figure 6).

**Figure 7:**
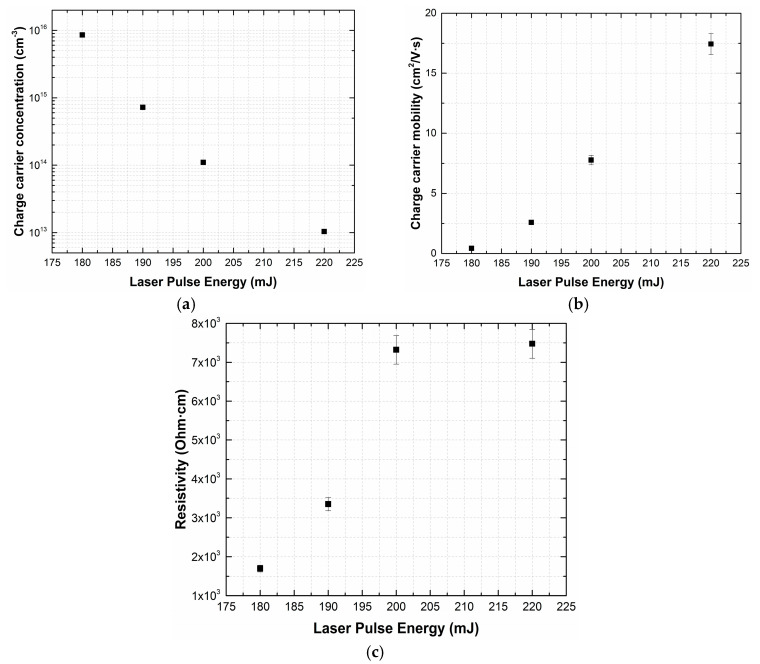
Dependences of the concentration (**a**) and mobility (**b**) of charge carriers, and resistivity (**c**) of LiNbO_3_ films on the laser pulse energy.

Increasing the laser pulse energy from 180 to 220 mJ results in a decrease of charge carrier concentration from 8.6 × 10^15^ to 1.0 × 10^13^ cm^−3^. The mobility of charge carriers increases from 0.43 up to 17.4 cm^2^/(V∙s), which can be associated with an increase in stoichiometry during heat and mass transfer in the transit space and a decrease in the defectiveness of films [43]. This indicates a transition from the evaporation mode to ablation (Figure 7). The structural changes of fabricated films deposited at different laser pulse energies can be attributed to the diffusion character of incident particles on the substrate [39,44].

**Figure 8:**
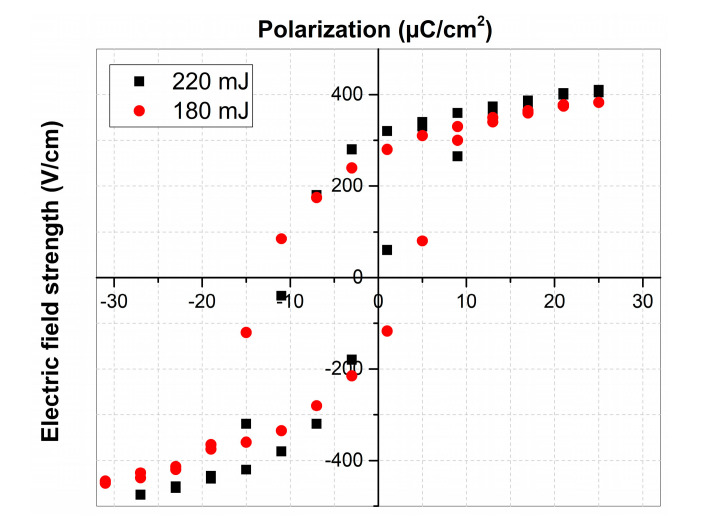
Polarization of the obtained LiNbO_3_ films.

Figure 8 shows the dependencies of the polarization of the fabricated LiNbO_3_ films on the electric field strength.

The dependencies of polarization on the field strength exhibit hysteresis, which characterizes these films as ferroelectric with spontaneous polarization.

### 3.2. CNT Growth

In addition, experimental studies of the sublayer material effect on the geometric parameters of CNTs were performed. Ni(10 nm)/V(20 nm)/Si and Ni(10 nm)/Cr(20 nm)/Si were chosen.

**Figure 9:**
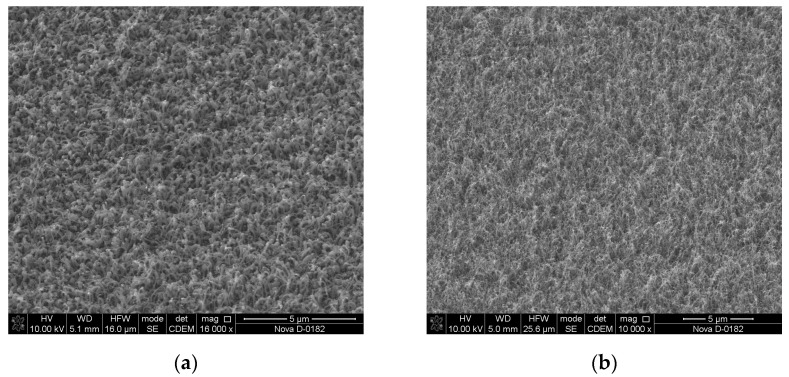
SEM images of carbon nanotubes grown on various sublayers (growth time 1 min): Ni/V/Si (**a**), Ni/Cr/Si (**b**).

Figure 9 shows SEM images of the obtained CNT arrays on the Ni/V/Si and Ni/Cr/Si structures.

**Figure 10:**
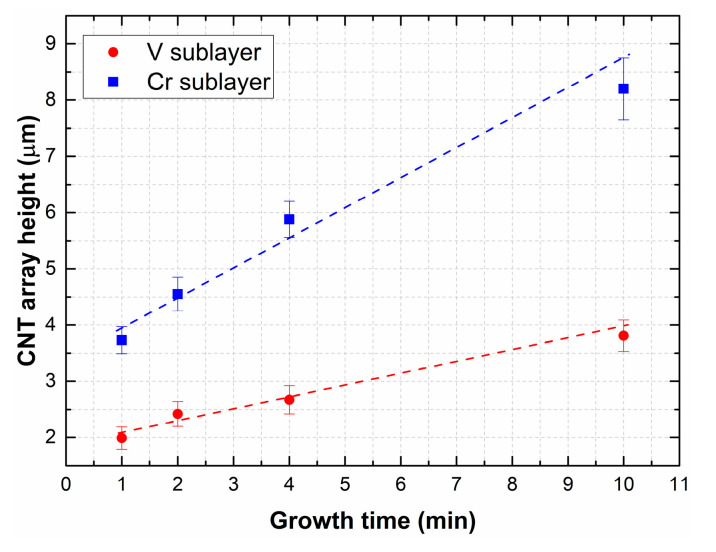
Dependence of the carbon nanotube (CNT) array height on the growth time, at the minimum plasma power.

Based on the obtained results, the influence of sublayer material (lower electrode) was established. The dependencies of the heights of CNTs on the growth times for various sublayer materials are shown in Figure 10.

It was found that the material of the lower electrode had a significant effect on the formation of CNTs. CNTs obtained at the same growth time on a sample with a Cr sublayer had a smaller diameter and a longer length compared to samples with a V sublayer. According to the results of experiments, an array of carbon nanotubes with the following parameters with a vertical orientation of 40–70 nm in diameter, and heights of 4.5–5 μm, was obtained. The density of the tubes in the array was around 3.68 × 10^5^ cm^−2^.

### 3.3. Piezoelectric Energy Harvester

**Figure 11:**
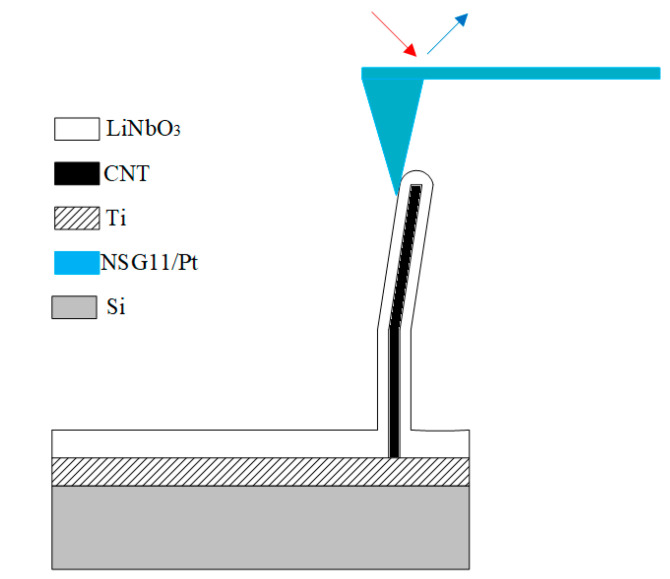
Schematic representation of the proposed nanogenerator.

Based on the obtained results, the architecture of the piezoelectric nanogenerator based on a hybrid carbon nanostructure was developed. As an active element it uses a CNT coated with a LiNbO_3_ film with ferroelectric and piezoelectric properties. Figure 11 shows a schematic representation of the developed nanogenerator.

The generated current were measurement measured by the force mode of the Ntegra probe nanolaboratory when the mechanical force were applied (Figure 11) [45]. The Si/Ti hybrid carbon nanostructure was used for measurements. An NSG11/Pt conducting AFM cantilever probe was used as the upper electrode. The pressing force of the NSG11/Pt AFM cantilever probe changed from 0 to 600 nN. The deformation of the structure was formed under the action of an AFM probe during force spectroscopy.

**Figure 12:**
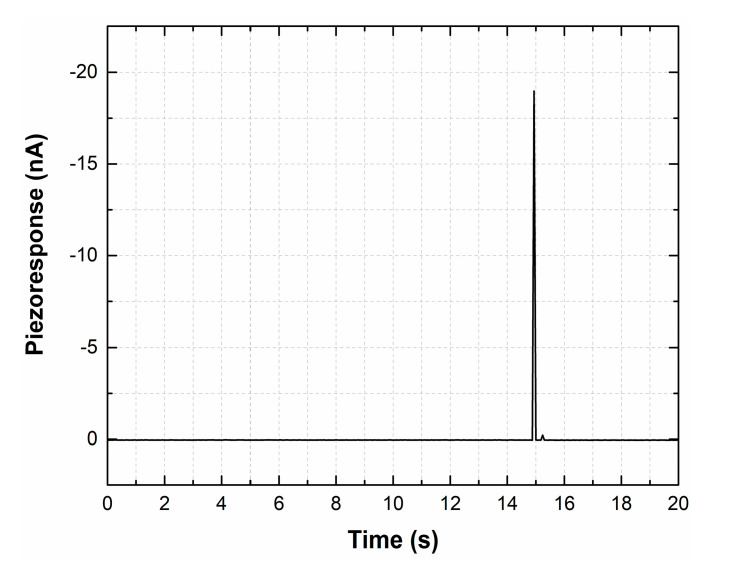
Piezoresponse of a nanogenerator to an applied mechanical action of a force of 600 nN.

The current value was reproducibly reduced to zero (Figure 12), with the removal of the mechanical impact.

The piezoelectric effect creates an electric field within the structure: a stretched part with positive strain creates a positive electrical potential, while a compressed part with negative strain creates a negative electrical potential. This effect is associated with the relative displacement of cations relative to anions. As a result, a potential difference is created at the top of the nanostructure and distributed over the surface of the structure. However, the base of the nanostructure remains electrically neutral.

**Figure 13:**
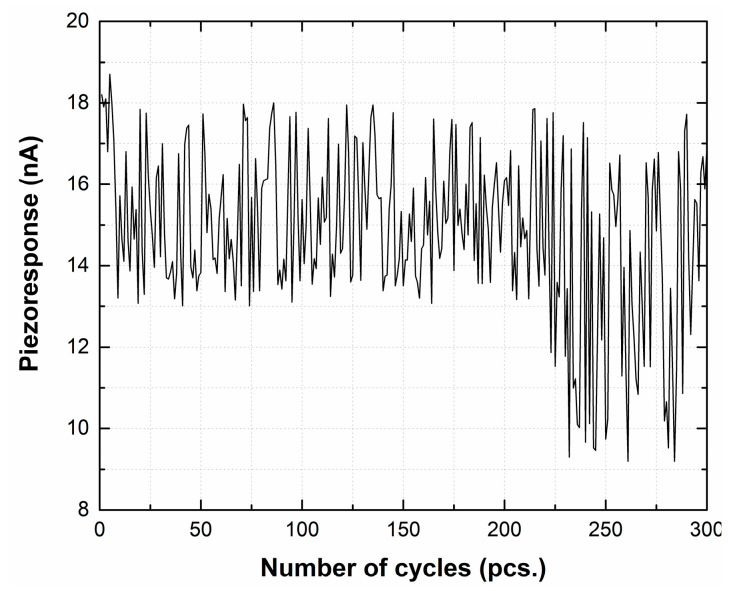
Dependence of the magnitude of the piezoresponse on the number of deforming cycles.

Figure 13 shows the results of studying the stability of the output parameters under multiple deforming cycles.

It was found that the hybrid-carbon-nanostructure-based nanogenerator generated a current of about 18 nA when mechanical stress of 600 nN was applied. In addition, under the influence of multiple deforming cycles, the nanogenerator retained its ability to generate current, despite the gradual degradation of its parameters that began after cycle 218. One of the possible ways to increase the stability of the nanogenerator parameters is the use of a polymeric material such as dimethicone (PDMS) as a flexible filler, which would perform a protective function without violating the elastic properties.

**Figure 14:**
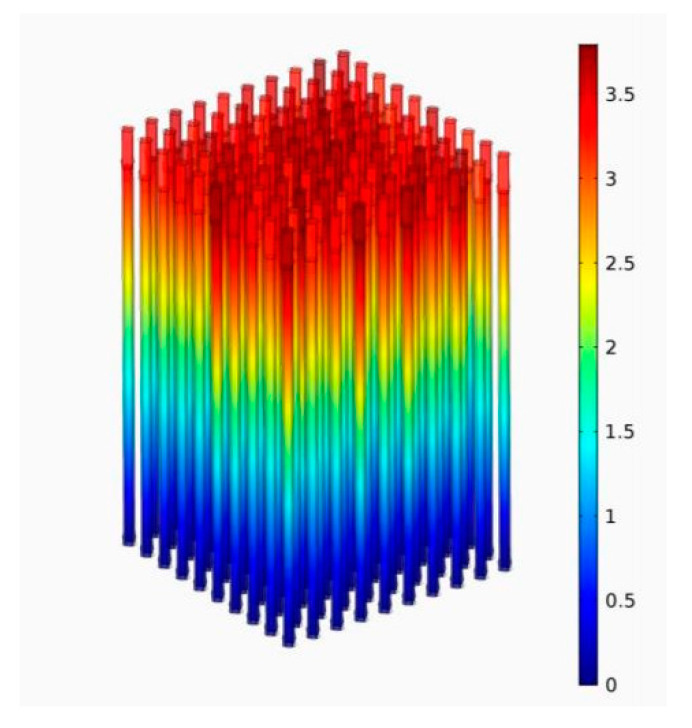
Distribution of electrical potential (V) across the piezoelectric transducer structure when exposed to a force of 1 N.

To test the integral effects associated with an increase in the generated energy with an increase in the number of hybrid carbon structures in the array, COMSOL Multiphysics 5.2 software (COMSOL Inc., Los Altos, CA, USA) was used. The established COMSOL model was based on the piezoelectricity, solid mechanics, electrostatics, and electric circuit modules. As a boundary condition, it was assumed that the bottom facet of the hybrid nanostructures was mechanically fixed and electrostatically neutral. In the same manner as in the experimental study, mechanical stress was applied from the side of the nanostructure array. We performed the estimated calculations of the parameters of the proposed structure and obtained the distribution of the electric potential in the piezoelectric transducer, which consisted of 100 hybrid carbon nanostructures. Figure 14 shows the calculated electrical potential distribution at mechanical stress of 1 N.

The generated voltage of the proposed piezoelectric transducer based on hybrid carbon nanostructures was at the level of modern transducers presented in the literature [46,47,48,49,50,51,52]. The active layers based on the hybrid carbon nanostructures make it possible to increase the generated voltage by a factor of 10 in comparison with a LiNbO_3_-based converter [52]. In addition, the absence of lead in the composition of the converter allows implementation of biocompatible energy harvesters on the same basis, which makes it possible to significantly expand the areas of their potential application, including for use as part of wearable electronic devices, medical equipment, and potential integration into clothes. The correctness of the model was verified by comparing the calculation results with the experimentally investigated characteristics of the nanogenerator. It was found that the calculated voltage peak generated by the piezoelectric structure was 3.5 V, which agrees with the experimental results.

## 4. Conclusions

Experimental studies of the parameters of lithium niobate films formed by PLD have shown that the laser pulse energy has an essential effect on the structure and properties of the films. The experiments have shown that films obtained in the range of laser pulse energies from 180 to 220 mJ exhibit ferroelectric properties. It was found that increasing laser pulse energy from 180 to 220 mJ resulted in decreasing charge carrier concentration of LiNbO_3_ films from 8.6 × 10^15^ to 1.0 × 10^13^ cm^−3^, while the mobility of charge carriers increased from 0.43 up to 17.4 cm^2^/(V∙s).

Based on the results obtained, the structure of the nanogenerator has been proposed. The proposed generator creates a current of 18 nA at mechanical stress of 600 nN. The conducted studies of the stability of the parameters of the hybrid-carbon-nanostructure-based nanogenerator have shown that the nanogenerator retains the ability to generate current despite the gradual degradation of its properties that significantly appears after 218 cycles. Based on the modeling it was found that an array of 100 nanostructures can generate a voltage of about 3.5 V.

The influence of laser pulse energy during PLD has been experimentally studied, which allowed LiNbO_3_ films with controlled parameters to be obtained. The obtained theoretical and experimental results enable fabrication of LiNbO_3_ films that can be used as a key element of the promising lead-free energy converters for “green” energy and IoT devices.
